# The learners' perspective on internal medicine ward rounds: a cross-sectional study

**DOI:** 10.1186/1472-6920-10-53

**Published:** 2010-07-09

**Authors:** Muhammad Tariq, Afaq Motiwala, Syed Umer Ali, Mehmood Riaz, Safia Awan, Jaweed Akhter

**Affiliations:** 1 Department of Medicine, Aga Khan University, Stadium Road, Karachi, Pakistan

## Abstract

**Background:**

Ward rounds form an integral part of Internal Medicine teaching. This study aimed to determine the trainees' opinions regarding various aspects of their ward rounds, including how well they cover their learning needs, how they would like the rounds to be conducted, and differences of opinion between medical students and postgraduates.

**Methods:**

A cross-sectional study was conducted on a total of 134 trainees in Internal Medicine, comprising medical students, interns, residents and fellows, who were asked to fill in a structured, self-designed questionnaire. Most of the responses required a rating on a scale of 1-5 (1 being highly unsatisfactory and 5 being highly satisfactory).

**Results:**

Teaching of clinical skills and bedside teaching received the lowest overall mean score (Mean ± SD 2.48 ± 1.02 and 2.49 ± 1.12 respectively). They were rated much lower by postgraduates as compared to students (p < 0.001). All respondents felt that management of patients was the aspect best covered by the current ward rounds (Mean ± SD 3.71 ± 0.72). For their desired ward rounds, management of patients received the highest score (Mean ± SD 4.64 ± 0.55), followed by bedside examinations (Mean ± SD 4.60 ± 0.61) and clinical skills teaching (Mean ± SD 4.50 ± 0.68). The postgraduates desired a lot more focus on communication skills, counselling and medical ethics as compared to students, whose primary focus was teaching of bedside examination and management. A majority of the respondents (87%) preferred bedside rounds over conference room rounds. Even though the duration of rounds was found to be adequate, a majority of the trainees (68%) felt there was a lack of individual attention during ward rounds.

**Conclusions:**

This study highlights important areas where ward rounds need improvement in order to maximize their benefit to the learners. There is a need to modify the current state of ward rounds in order to address the needs and expectations of trainees.

## Background

Lectures and presentations, conferences, discussions, and self-reading all contribute to the learning and grooming of in-training doctors and medical students. However a valuable tool for learning, particularly in the medicine specialties, are the teaching ward rounds, particularly those conducted by the attendings. They form the corner stone of Internal Medicine teaching [1-6]. They provide an avenue where senior physicians can pass down their experience to the learners, teach relevant topics, update trainees with current literature, and help in the application of theoretical knowledge into direct patient care. Ward rounds represent a complex task requiring not only medical knowledge but also communication skills, clinical skills, teaching skills, patient management skills and team-work skills [7-9]. They constitute the most effective means of providing learners with various tasks and roles that they would have to perform as doctors, including managing a team, the doctor-patient relationship, counselling of patients and their families, and breaking bad news.

Several authors have tried to identify barriers to the full utilization of the potential benefits of ward rounds. These include time constraints, faculty attitude, knowledge and skill, lack of respect for the patient and over reliance on technology [10]. According to another study, the most important detractors to the success of rounds include a disrespectful attitude by attendings, and rounds that are too long or too short [1]. Time constraints have also been shown to be an important factor, arising from pressure to see more and more patients, shortened hospital stays, and increased demands for documentation [11,12]. In our study, we aimed to determine the trainees' opinions regarding various aspects of their "attendings' ward rounds," how well these rounds cover their learning needs, how they would like the rounds to be conducted, and differences of opinion between medical students and postgraduates.

## Methods

### Study design and location

We conducted a cross-sectional study at the Aga Khan University Hospital (AKUH), Karachi (Pakistan), during the period of March 2008 to May 2008. AKUH is one of the major tertiary care hospitals in the private sector of Karachi (Pakistan), having an operational strength of 545 beds. It is also a centre for undergraduate and postgraduate teaching and ranks as a leading teaching medical institute in the country with international recognition and JCI (Joint Commission International) accreditation.

### Study sample

Our study subjects were post-graduate trainees (interns, residents and fellows) who had rotated through or were currently rotating through Internal Medicine at our institute. They were included only if they had spent at least 3 months in Internal Medicine. Fellows who had completed their Internal Medicine residencies within the last 3 years were also included. All postgraduates who had been through Internal Medicine more than 3 years ago were omitted from the study. Medical students in their clinical years (third, fourth and fifth years) were also included in the study. Students of year 3 were only included if they had completed their Internal Medicine rotation. The target population was based on convenience sampling. Sample size was of 134.

### Structure of "attending rounds"

During Internal Medicine rotations at our institute, daily teaching rounds are conducted by the attending who is leading that team for that particular month. Typically our "attending rounds" teams comprise, apart from one attending, one senior resident (Resident year 3 or 4), two junior residents (Resident year 1), one intern, two final year (year 5) medical students, and one to two Year 3 medical students. In our study we have focused entirely on these "attending ward rounds."

### Study questionnaire

The sample population was asked to fill in a self-administered questionnaire. This questionnaire was designed based on our experience and brain storming with further additions and amendments made using previous literature. The questionnaire included a multitude of questions which were intended to assess the participants' opinion of their current ward rounds and how they would like the rounds to be conducted. In the questionnaire, the participants were asked to rate, on a scale of 1 - 5, various aspects of their current ward rounds and the ward rounds that they desire. These aspects included teaching of clinical skills and medical knowledge, patient management, bioethics, communication skills and a few other attributes. The questionnaire also included questions on frequency and duration of rounds, number of participants who attended these rounds, and whether they were conducted at the bedside or in conference rooms. In addition, other questions assessed lack of individual attention, need for separate teaching faculty, and other disciplines that could be involved during rounds. Additional file 1 shows the questionnaire used in our study.

### Data collection

The questionnaire was initially pretested on a convenience group of 10 participants and then improved accordingly. The final questionnaire had a total of 35 questions and the pretesting showed that the questionnaire took approximately 10-15 minutes to complete. Subsequently, the questionnaire was administered to the participants by trained medical students, who were also available to provide assistance in filling the form. However, the students were instructed not to influence the participants' responses in any possible way. Verbal consent was obtained from all subjects prior to administration of the questionnaire. In order to maintain complete confidentiality no names were recorded on the questionnaire. Prior approval of the hospital administration was also obtained before beginning the survey. The study was approved by the Ethical Review Committee of the Department of Medicine at the Aga Khan University Hospital (AKUH), Karachi, Pakistan.

### Analysis

Data was entered and analyzed using standard biostatistics software package (SPSS). The data was compiled and tabulated, and comparisons were made between the responses of medical students and post-graduate trainees. Associations were assessed using Chi-square test for categorical data and t-test for continuous data, whichever was applicable. All the tests were carried out at 5% level of significance.

## Results

### Respondents

A total of 134 respondents filled out the questionnaire. Eighty-two (61.2%) were males and 52 (38.8%) were females. Almost half of all respondents were medical students, being 68 in number (50.7%), and the remaining 66 (49.3%) were postgraduate trainees. Among the postgraduates, 11 (16.7%) were interns, 49 (74.2%) were residents and 6 (9.1%) were fellows.

### General outline of responses

All the participants thought that teaching of patient management was the aspect best covered by ward rounds (rating of Mean ± SD 3.71 ± 0.72). The aspects rated most unsatisfactory were teaching of clinical skills (Mean ± SD 2.48 ± 1.02) and bedside examination (Mean ± SD 2.49 ± 1.12). When assessing the desired qualities of an ideal ward round, the highest mean rating was for teaching of management of patients (Mean ± SD 4.64 ± 0.55), followed by bedside examination (Mean ± SD 4.60 ± 0.61) and then teaching of clinical skills (Mean ± SD 4.50 ± 0.68).

### Comparison between medical students and postgraduate trainees

When these ratings were analyzed separately for medical students and postgraduates, they were found to differ in terms of both their opinions and expectations. Table 1 compares the opinions of medical students and postgraduates regarding various aspects of their 'current' teaching rounds. Table 2 compares these same aspects that medical students and postgraduates desire for their ideal ward rounds.

**Table 1:** Comparison between medical students and postgraduates about how much their CURRENT 'Internal Medicine' ward rounds cover the following competencies.

	Students (n = 68)	Postgraduates (n = 66)	
**Competencies**	**Mean ± SD**	**Mean ± SD**	p value*

Conveying medical knowledge	2.94 ± 0.97	2.81 ± 0.77	0.405

Teaching clinical skills	2.79 ± 0.95	2.16 ± 1.0	<0.001

Professional attitude	3.41 ± 0.93	3.26 ± 0.94	0.374

Communication skills	3.23 ± 0.88	3.45 ± 0.76	0.139

Clinical problem solving ability	3.36 ± 0.86	3.14 ± 0.81	0.123

Presentation skills	3.50 ± 0.92	3.0 ± 0.98	0.003

Approach towards patients	3.35 ± 0.93	3.35 ± 0.83	0.983

Management of patients	3.63 ± 0.78	3.80 ± 0.64	0.171

Ability to discuss problems logically	3.20 ± 0.89	3.25 ± 0.89	0.759

Medical ethics	2.95 ± 1.04	3.15 ± 1.03	0.266

Counseling	3.34 ± 0.97	3.57 ± 1.0	0.178

Bedside examination	2.79 ± 1.07	2.17 ± 1.10	0.001

Managerial skills	2.51 ± 0.98	2.87 ± 1.06	0.044

Leadership skills	2.70 ± 0.96	3.09 ± 0.99	0.025

(Competencies rated on a scale of 1 - 5)

*Statistical test conducted: Independent sample t-test

**Table 2:** Comparison between medical students and postgraduate about how much their ward rounds should cover the following competencies.

	Students (n = 68)	Postgraduates (n = 66)	
**Competencies**	**Mean ± SD**	**Mean ± SD**	p value*

Conveying medical knowledge	4.43 ± 0.74	4.28 ± 0.92	0.311

Teaching clinical skills	4.49 ± 0.68	4.50 ± 0.68	0.895

Professional attitude	4.34 ± 0.80	4.37 ± 0.68	0.845

Communication skills	4.05 ± 0.86	4.43 ± 0.65	0.008

Clinical problem solving ability	4.31 ± 0.83	4.55 ± 0.60	0.068

Presentation skills	4.26 ± 0.86	4.48 ± 0.57	0.104

Approach towards patients	4.31 ± 0.74	4.61 ± 0.52	0.011

Management of patients	4.59 ± 0.58	4.71 ± 0.52	0.226

Ability to discuss problems logically	4.34 ± 0.79	4.50 ± 0.74	0.256

Medical ethics	4.01 ± 1.04	4.54 ± 0.71	0.002

Counseling	4.24 ± 0.76	4.57 ± 0.73	0.014

Bedside examination	4.66 ± 0.53	4.53 ± 0.68	0.270

Managerial skills	3.89 ± 1.03	4.18 ± 0.82	0.097

Leadership skills	3.98 ± 0.95	4.42 ± 0.80	0.007

(Competencies rated on a scale of 1 - 5)

*Statistical test conducted: Independent sample t-test

**Figure 1:**
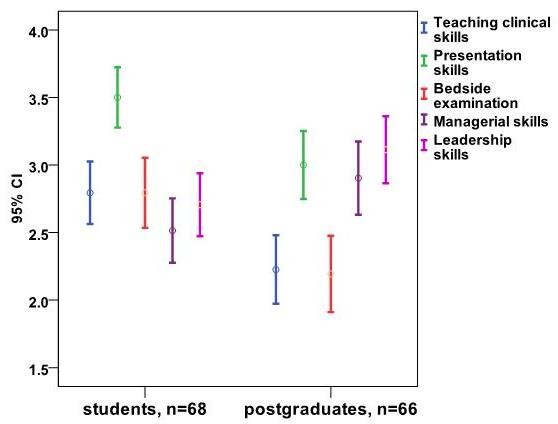
**How much do your current 'medicine' ward rounds cover the following competencies? **Comparison between medical students and postgraduates.

Figure 1 is a graphical representation of the data from Table 1, where the ratings of both medical students and postgraduates for their "current" ward rounds are distinctly evident.

### Other attending ward round characteristics

**Figure 2:**
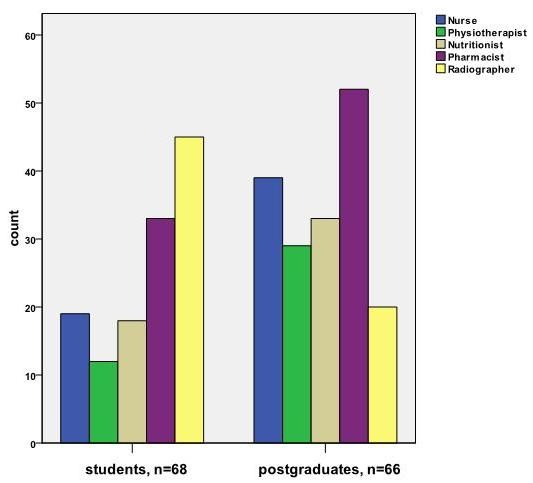
**Multidisciplinary ward rounds? **Other disciplines that should be part of the Internal Medicine ward round team.

Out of the total participants, 113 (84.3%) were of the view that ward rounds should be multi-disciplinary in order to enhance learning and efficiency. Other disciplines which were presented as options to be included as part of the ward round team included Nursing, Physiotherapy, Nutritionist, Pharmacy and Radiology. The responses of both medical students and postgraduates regarding each of these disciplines are shown in Figure 2.

Seventy four (55.2%) of the respondents thought the ward rounds were mainly service-oriented, only 6 (4.5%) thought they were teaching oriented whereas 54 (40.3%) thought they were balanced service/teaching oriented. However, when asked how they should be, 93 (69.4%) wanted the rounds to be balanced service/teaching oriented, 40 (29.9%) wanted them to be purely teaching oriented and none of the participants wanted them to be only service oriented. When asked where the ward rounds were mainly conducted, 132 (98.5%) of the respondents said they were conducted primarily on patient bedsides, whereas only 2 (1.5%) said they were done in conference rooms. In answer to where the rounds should be conducted, 117 (87.3%) wanted the rounds to be conducted on bedsides and 17 (12.7%) wanted them in conference rooms.

### Adequacy of time and individual attention

The average time spent per patient on rounds was found to be 12 minutes (SD ± 7 minutes) whereas the ideal average time that should be spent per patient came out to be 14 minutes (SD ± 6 minutes). The total number of members during the rounds was found to be between 4 and 20, with a mean of 8 members. Sixty-eight percent of the respondents felt there was a lack of individual attention during the ward rounds.

### Comparison between current and ideal ward rounds

**Figure 3:**
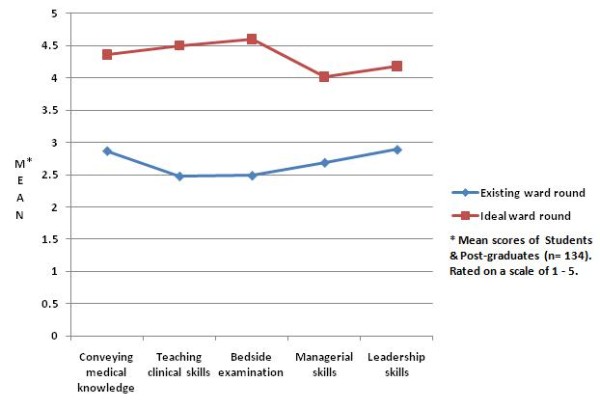
**Major differences observed between current and ideal ward rounds**.

Figure 3 demonstrates areas where a large difference was found in opinions of the learners between 'current' and 'ideal' ward rounds.

## Discussion

Our study shows that from a trainee's perspective, teaching of clinical skills is the weakest aspect of the current ward rounds. This is interesting considering the number of discussions in recent times regarding the deterioration of clinical skills in young doctors. It is widely believed that there is a current trend towards increased dependence on investigations and imaging, and a declining emphasis on clinical judgement, which is adversely affecting the clinical skills of the newer generation of physicians [13]. The fact has been reiterated time and again that ward rounds are the most important tool for teaching of clinical skills [2,10,14,15]. Despite importance of bedside teaching, which takes place mainly during teaching rounds, its frequency is progressively decreasing [16]. Nikendei et al. in their study observed that final year students had severe deficits in their ward round skills, with the deficits predominantly in focused physical examination, chart reviewing, prescriptions and documentation [17,18]. One of the important factors influencing these deficits is lack of supervision [19-21]. This lack of supervision arises especially where trainees conduct independent patient examinations, but do not have opportunities to conduct supervised examinations. Comparative analysis in our study showed that such clinical skills teaching was rated much lower by post graduates as compared to medical students. This often occurs due to the tendency of most faculty members to focus such basic skills teaching more on medical students, assuming that the residents at their stage would have mastered those techniques.

When questioned regarding the qualities of the desired ward rounds, teaching of patient management was rated the highest, followed by teaching of clinical skills and bedside teaching. Management of patients was also rated on the top in the current ward rounds category. This is an important finding from our study. It shows that the participants attach a great deal of importance to the management of patients, making it one of the most essential aspects of the ideal ward round. It is very satisfying to note that this aspect has also been rated highest in the current round category, which clearly shows the participants are satisfied with this aspect of teaching in their ward rounds, and that this aspect does currently receive the importance that it demands.

One area where a significant difference was observed between medical students and post-graduates in the qualities they described for their desired ward rounds was in teaching of medical ethics and patient counselling. This was rated much higher by the post-graduates. We feel the reason for this is that at the post-graduate training level, doctors are regularly faced with a number of ethical questions and situations requiring effective communication skills. We also found that the postgraduate certificate examination had a much greater proportion devoted to ethics and patient counselling, making this aspect of teaching even more important for them. When comparing the current ward rounds with the desired ward rounds for both students and postgraduates, we found that a large difference was observed in the conveying of medical knowledge, teaching of clinical skills and bedside examination, as well as in teaching of managerial and leadership skills. Thus these represent aspects which, in the opinion of the learners, have the largest room for improvement and may be used as starting points in the long-term improvement of ward rounds.

Previous studies have stated that ward round teaching is an essential tool of training but it is significantly under-utilized [10]. Nair et al in their study reported that only 48% of the learners reported they had been given adequate bedside teaching during their undergraduate training [14]. Various studies in the past have also tried to identify barriers to the full utilization of the potential benefits of teaching ward rounds, some of which have been mentioned above. Many authors have also suggested potential solutions to the barriers. A study by Castiglioni et. al. to assess the perception of residents and interns regarding successful rounds describes approachability of the attendings, their enthusiasm for teaching, involving learners in the teaching process and establishing their goals/expectations as the most important success factors. A study conducted to explore the faculty's perception of barriers to effective bedside teaching reports that declining clinical skills and teaching values were some of the major barriers, as well as intense performance pressure arising from the belief that the teachers should possess an almost unattainable level of diagnostic skill. They recommend training of the clinical teachers, their reassurance, and establishing a conducive learning environment to mitigate such barriers [22].

When assessing the approximate being time spent on patient bedsides, we found an average time per patient of 12 minutes, while the suggested ideal average time was found to be 14 minutes. There have been studies which have reported much lower average times [23-25]. However, these studies had actually recorded the exact amount of time and thus we cannot make a very reliable quantitative comparison with our results. Albeit, we are reluctant in recommending lengthier rounds in an attempt to improve their effectiveness since a number of previous studies describe brevity and focused discussion as an important success factor [1,24]. In one study, 60% of learners preferred new patient presentations of less than 5 minutes per patient [26]. In another study, 75% of attendings and 89% of residents desired new cases to be presented in 5 minutes or less [27]. More importantly, a majority of the participants in our study felt there was a lack of individual attention during ward rounds, which can be attributed to the large number of members in ward round teams. Seventy-five percent of the learners in our study also thought that there a need for separate teaching faculty for clinical and bedside teaching.

We noted a preference for bedside rounds compared to conference room rounds in our participants. This has been a subject for much debate and contradiction in previous literature [26,28]. Although patients predominantly prefer bedside rounds carried out in their presence, since that would make them feel more involved in their plan of care, the learners have generally been shown to prefer rounds, particularly case presentations, away from the patients [12,27,29]. Wang-Cheng et. al. reached a similar conclusion, and described that the attendings were evenly divided in preference, with more of the younger staff preferring the conference room setting [26]. In view of these, it is interesting to note that eighty seven percent of our study population wanted rounds to be conducted at bedsides rather than in conference rooms. However, most of them (91.8%) thought there was a need for post-round group discussions/tutorials.

In order to enhance the benefit of teaching rounds, and to ensure both faculty and trainee satisfaction, significant importance has to be given to preparing and planning out the goals prior to the rounds and orienting the trainees with those goals. Equally important is challenging the learner's thinking with questions and gentle correction without any humiliation, and also observing their clinical skills. At the conclusion of the rounds, it is valuable to summarize the teachings of that round, and to leave room for clarifications, discussions and assigning further reading [30].

### Limitations of the study

Our study had a sample size of 134 individuals, which may not represent individual views of all the trainees. However most such studies have had similar or lower sample sizes and we believe our sample adequately represents the overall population of medical students and postgraduates at our institute. Amongst the postgraduate group, there was an under-representation of fellows, who comprised less than ten percent of the postgraduates. We did not separately analyze the views of interns, residents and fellows, although they may have had some differences in their opinions and expectations. Similarly, medical students in third year and final year were grouped together in their views. However for concrete results and initial steps for a change, we believe it is fairly reliable to place all the medical students in one group and all the postgraduates into the other. This has given us a fairly good idea of what each group as a whole thinks about and expects from their ward rounds. We also did not separately analyze the opinions of the participants based on their gender, although there could have been a possible difference between the responses of males and females.

Even though we had omitted from our study all postgraduates who had been through Internal Medicine more than 3 years ago, we still realize that 3 years is a considerably long period. Thus, even with postgraduates who had been through Internal Medicine less than 3 years ago, accuracy of recall may have been a problem and could have led to a possible recall bias. We did not pick out a random sample and our study sample was based on simple convenience sampling. Thus we also cannot rule out the possibility of a selection bias. For comparisons between students and postgraduates, a number of t-tests were carried out. However, no Bonferroni correction was performed, which may have possibly led to some degree of inaccuracy.

External validity of our study may also have been a limitation since our study was centered only at one tertiary care teaching hospital in Karachi. The results may not have been generalizable to the numerous other medical institutes in Pakistan. However, our institute is considered amongst the top institutes in Pakistan in terms of education and research by official Higher Education Commission rankings [31]. This leads us to believe that the state of the remaining institutes may be similar or worse and any short comings in ward rounds and teaching that we discovered would most likely be even more prevalent at other institutes. Ideally, the structuring of the ideal ward rounds should take into account the perspective of the patients as well. However we decided to focus only on the students and postgraduates to provide a clear and focused perspective of the 'learners' who are part of the rounds. We felt the patients' perspective could best be described through a separate study.

## Conclusions

Our study quiet vividly points to certain areas of ward rounds that need particular attention in order to maximize their benefit to the learners. It shows that the teaching of clinical skills and bedside examination are avenues that are of great importance to the learners but are not being adequately addressed with the current state of our rounds. Even though the time being spent per patient may be close to appropriate, participants feel there is a lack of individual attention during the rounds. Based on their views, we recommend smaller teams, a more organized approach to teaching, with possibly a separate clinical teaching faculty, and rounds at bedsides with post-round conference room discussions.

In view of the information gathered from this study, as well as using the opinions of the faculty themselves, we plan to form a set of guidelines to improve the efficiency of ward rounds and increase their acceptability for both medical students and postgraduates. Subsequently, we plan to undertake a follow up study gathering the same information from a similar study population. This would provide a reliable estimation/interpretation of how the guidelines based on our study are able to improve ward round satisfaction. Based on the interpretations, our guidelines can be more generalized to be adopted in other departments including surgery, pediatrics, obstetrics and gynecology at our institute as well as at other institutions.

## Competing interests

The authors declare that they have no competing interests.

## Authors' contributions

MT conceived the idea for the study and contributed in making the questionnaire and writing the manuscript. He also supervised conducting the study. AM contributed in making the questionnaire, collected and analyzed the data and wrote the manuscript.

SUA also participated in data collection, analysis and helped in writing the manuscript. MR helped in conceiving the idea. SA worked on data analysis and provided statistical help. JA reviewed the manuscript. All authors read and approved the final manuscript.

## Pre-publication history

The pre-publication history for this paper can be accessed here:

http://www.biomedcentral.com/1472-6920/10/53/prepub

## Supplementary Material

Additional file 1 **Study questionnaire**. The questionnaire used in our study. Click here for file
