# Supplementary material for: The learners' perspective on internal medicine ward rounds: a cross-sectional study
Source: BMC Med Educ. 2010 Jul 9;10:53. doi: 10.1186/1472-6920-10-53 (PMC2912319; doi:10.1186/1472-6920-10-53)
Supplement: Additional file 1 — Study questionnaire. The questionnaire used in our study. [file 1472-6920-10-53-S1.DOC]

# Internal Medicine ward rounds; a learner’s perspective

# **Name (optional)**

# **Gender: M F**

| **Intern / Resident / Fellow** | |
| --- | --- |
| Department |  |
| Year of residency/  fellowship | Intern R1 R2  R3 R4 R5  F1 F2 F3 |
| Institute of training | AKUH |
| **Graduation** | |
| Year of graduation |  |
| Institute of graduation |  |

| **Student** | |
| --- | --- |
| Year of medical school | 3 4 5 Graduated |
| Institute of training | AKU-Medical college |

| S.No | Competencies | How much do your CURRENT ‘medicine’ ward rounds cover the following competencies? | | | | |
| --- | --- | --- | --- | --- | --- | --- |
|  |  | **1 is lowest, 5 is highest** | | | | |
| 1 | Conveying medical knowledge | 1 | 2 | 3 | 4 | 5 |
| 2 | Teaching clinical skills | 1 | 2 | 3 | 4 | 5 |
| 3 | Professional attitude | 1 | 2 | 3 | 4 | 5 |
| 4 | Communication skills | 1 | 2 | 3 | 4 | 5 |
| 5 | Clinical problem solving ability | 1 | 2 | 3 | 4 | 5 |
| 6 | Presentation skills | 1 | 2 | 3 | 4 | 5 |
| 7 | Approach towards patients | 1 | 2 | 3 | 4 | 5 |
| 8 | Management of patients | 1 | 2 | 3 | 4 | 5 |
| 9 | Ability to discuss problems logically | 1 | 2 | 3 | 4 | 5 |
| 10 | Medical ethics | 1 | 2 | 3 | 4 | 5 |
| 11 | Counseling | 1 | 2 | 3 | 4 | 5 |
| 12 | Bedside examination | 1 | 2 | 3 | 4 | 5 |
| 13 | Managerial skills | 1 | 2 | 3 | 4 | 5 |
| 14 | Leadership skills | 1 | 2 | 3 | 4 | 5 |

| How much do you **THINK** your ward rounds should cover the following competencies? | | | | |
| --- | --- | --- | --- | --- |
| **1 is lowest, 5 is highest** | | | | |
| 1 | 2 | 3 | 4 | 5 |
| 1 | 2 | 3 | 4 | 5 |
| 1 | 2 | 3 | 4 | 5 |
| 1 | 2 | 3 | 4 | 5 |
| 1 | 2 | 3 | 4 | 5 |
| 1 | 2 | 3 | 4 | 5 |
| 1 | 2 | 3 | 4 | 5 |
| 1 | 2 | 3 | 4 | 5 |
| 1 | 2 | 3 | 4 | 5 |
| 1 | 2 | 3 | 4 | 5 |
| 1 | 2 | 3 | 4 | 5 |
| 1 | 2 | 3 | 4 | 5 |
| 1 | 2 | 3 | 4 | 5 |
| 1 | 2 | 3 | 4 | 5 |

# Could the ward rounds be multi disciplinary? (See next question for clarification) Y N

**If yes, what other disciplines do you think should be a part of the Internal Medicine ward rounds’ team?**

Nursing

Physiotherapy

Nutritionist

Pharmacy

Radiology

# Are your ward rounds?

Service oriented

Teaching oriented

Balanced Teaching/ service

**Do you want your ward rounds to be?**

Service oriented

Teaching oriented

Balanced Teaching/ service

# How often do consultants take ward rounds?

Daily Alternate day

Twice a week Once a week

Biweekly Others ________

**Where are your ward rounds conducted?**

Bedside

Conference rooms

**Where do you prefer your ward rounds to be conducted?**

Bedside

Conference room

**In general, what is the average time spent on each patient? _______** minutes

**What, in your opinion, should be the ideal time spent on each patient? ________** minutes

**In general, how many members round with the attending (consultant) during the rounds (including instructors, fellows, residents, interns and students)?**

**________**

**Do you think there is a lack of individual attention during ward rounds? Y N**

**Do you have small group sit down tutorials during or after ward rounds? Y N**

**Is there a need of small group sit down tutorials during or after ward rounds? Y N**

**Do you have a separate teaching faculty for clinical methods and bedside teaching? Y N**

**Is there a need of separate teaching faculty for clinical and bedside teaching? Y N**

**Do you wash your hands after examining every patient? Y N**

**What is your opinion on the general attitude of the consultants towards members of their teams? Rate in a scale of 1 to 5 (1 is extremely unsatisfactory, 5 is extremely satisfactory):**

**____________**

**Any other comments ___________________________________________________________**

**______________________________________________________________________________**
